# Quantitative Values from Synthetic MRI Correlate with Breast Cancer Subtypes

**DOI:** 10.3390/life12091307

**Published:** 2022-08-25

**Authors:** Toshiki Kazama, Taro Takahara, Thomas C. Kwee, Noriko Nakamura, Nobue Kumaki, Naoki Niikura, Tetsu Niwa, Jun Hashimoto

**Affiliations:** 1 Department of Diagnostic Radiology, Tokai University School of Medicine, Isehara 259-1193, Japan; 2 Department of Biomedical Engineering, Tokai University School of Engineering, Hiratsuka 259-1207, Japan; 3 Department of Radiology, Nuclear Medicine, and Molecular Imaging, University Medical Center Groningen, 9700 RB Groningen, The Netherlands; 4 Department of Pathology, Tokai University School of Medicine, Isehara 259-1193, Japan; 5 Department of Breast Oncology, Tokai University School of Medicine, Isehara 259-1193, Japan

**Keywords:** magnetic resonance imaging, breast neoplasms, receptors, estrogen, quantitative values

## Abstract

The purpose of this study is to correlate quantitative T1, T2, and proton density (PD) values with breast cancer subtypes. Twenty-eight breast cancer patients underwent MRI of the breast including synthetic MRI. T1, T2, and PD values were correlated with Ki-67 and were compared between ER-positive and ER-negative cancers, and between Luminal A and Luminal B cancers. The effectiveness of T1, T2, and PD in differentiating the ER-negative from the ER-positive group and Luminal A from Luminal B cancers was evaluated using receiver operating characteristic analysis. Mean T2 relaxation of ER-negative cancers was significantly higher than that of ER-positive cancers (*p* < 0.05). The T1, T2, and PD values exhibited a strong positive correlation with Ki-67 (Pearson’s r = 0.75, 0.69, and 0.60 respectively; *p* < 0.001). Among ER-positive cancers, T1, T2, and PD values of Luminal A cancers were significantly lower than those of Luminal B cancers (*p* < 0.05). The area under the curve (AUC) of T2 for discriminating ER-negative from ER-positive cancers was 0.87 (95% CI: 0.69–0.97). The AUC of T1 for discriminating Luminal A from Luminal B cancers was 0.83 (95% CI: 0.61–0.95). In conclusion, quantitative values derived from synthetic MRI show potential for subtyping of invasive breast cancers.

## 1. Introduction

Breast cancer is the most frequently diagnosed malignancy and the leading cause of cancer-related death among women [1]. Breast cancer is a heterogeneous disease with a high degree of diversity with regards to the risk of therapeutic resistance and disease progression [2]. Therefore, the need for individualized management is widely accepted [2,3]. In addition to the traditional parameters, such as tumor size, grade, and lymph node status, the immunohistochemical-based classification is widely used [2,3]. Immunohistochemical assessments for tumor subtyping concern estrogen receptor (ER) and human epidermal growth factor receptor 2 (HER2) expressions, and proliferation according to the Ki-67 labeling index (Ki-67) [2,3].

The presence of ER or progesterone receptor (PgR) is a predictive marker of the long-term outcome and response to hormonal therapy [2,3]. Luminal-type (hormone receptor–positive) breast cancer is the most frequent subtype and is itself divided into two subtypes, namely Luminal A and Luminal B. Luminal A is defined as a low-proliferation subtype (Ki-67 < 14), whereas Luminal B is defined as a high-proliferation subtype (Ki-67 ≥ 14) [2,3,4]. Luminal A is not responsive to chemotherapy. Luminal B may be amenable to chemotherapy as well as endocrine therapy [2,3]. For hormone receptor-negative breast cancer, chemotherapy, frequently combined with molecular-targeted drugs, may be useful [2,3]. Since these receptor expressions can change during treatment [5,6], noninvasive assessment of receptor status, especially in recurrent lesions, may improve personalized treatment. As such, non-invasive assessment of Ki-67 may help in selecting appropriate adjuvant therapy.

Many researchers have explored the relationship between breast cancer subtypes and imaging findings. Breast magnetic resonance imaging (MRI) plays an important role in the detection, diagnosis, and staging of breast cancers [7]. Quantitative MRI methods, such as diffusion-weighted imaging (DWI) with apparent diffusion coefficient (ADC) measurements, have been used to discriminate breast cancer subtypes. However, recent meta-analyses reported that there is no significant correlation between the apparent diffusion coefficient (ADC) and Ki-67, and that there is a significant overlap in ADCs between breast cancer subtypes [8,9].

The recently developed technique of synthetic MRI allows for quantitative analysis of T1 relaxation, T2 relaxation, and proton density (PD), as well as generating various image contrasts using the information from one single scan [10,11,12]. Several investigators have reported that aggressive breast cancers tend to have high signal intensity on T2-weighted images [13,14,15,16,17,18,19,20,21]. We hypothesized that quantitative MRI parameters, such as T2 relaxation and proton density, correlate to the tumor grade of breast cancers.

The purpose of this study was twofold: to correlate quantitative T1, T2, and PD values obtained using synthetic MRI with tumor grade of breast cancers, and to determine if these quantitative values obtained using synthetic MRI can differentiate subtypes of breast cancers.

## 2. Materials and Methods

### 2.1. Subjects

The prospective study protocol was approved by the Institutional Review Board for Clinical Research, Tokai University (ref. 19R-182). Between November 2020 and February 2021, patients with histologically proven invasive breast carcinoma, a tumor size larger than 15 mm on ultrasound, and who were referred for pretreatment evaluation, underwent breast MRI. Thirty consecutive patients were invited in this study and written informed consent was obtained from all participants.

### 2.2. MR Image Acquisition

Studies were performed on a 1.5 T scanner (Ingenia, Philips Healthcare, Best, The Netherlands) using a 16-channel breast-phased array coil. Images were acquired in the prone position and in axial orientation to cover the whole breasts. Participating patients underwent synthetic MRI in addition to routine clinical MRI sequences. In synthetic MRI, quantification of T1 and T2 relaxation rates, as well as PD, was performed using the QRAPMASTER pulse method sequence, which is a multi-slice, multi-echo, and multi-saturation delay acquisition sequence [11]. Two sets of echo times (TEs) and 4 sets of delay times were used to generate 8 complex images in each section in order to quantify T1, T2, and PD. The TEs were 12.5 and 100 msec, the delay times were 151, 604, 2115, and 4382 msec, and the repetition time (TR) was 4462 msec. Thirty slices were acquired, voxel size was 1.42 × 1.92 × 4.00 mm^3^, and total acquisition time was 3:40 min. Synthetic images were created using SyMRI StandAlone software (SyntheticMR AB). Following synthetic MRI, routine T1-weighted images (TR/TE, 600/12 msec), STIR (TR/TE/TI, 3500/90/160 msec), DWI using single-shot echo planar imaging (b values of 0 and 1500 s/mm2; number of excitations, 1 and 14; TR/TE/TI, 6407/86/180 msec; field of view (FOV), 340 mm; matrix, 112 × 112; section thickness, 4 mm), and three-dimensional (3D) fat-suppressed dynamic contrast-enhanced MRI (TR/TE, 4.0/2.1 msec; flip angle, 15°; FOV, 340 mm; matrix, 352 × 352; slice thickness, 1 mm; intersection gap, 0 mm) were obtained. Dynamic scans were performed before and 4 times after the rapid injection of a bolus of 0.1 mmol/L of gadolinium-based contrast medium per kilogram of body weight.

### 2.3. Image Analysis

MR images were evaluated by a board-certified radiologist with 20 years of experience in breast MRI. Dedicated analysis software for synthetic MRI automatically calculated a value of R1 (i.e., 1/T1), R2 (i.e., 1/T2), and PD, for all pixels in the ROI. The PD level of pure water was set at 100%. Synthetic T1-weighted images (TR/TE, 600/10 msec) were made for measurement purposes. For measurement of T1, T2, and PD, the radiologist selected the slice that showed the largest surface of the tumor on contrast-enhanced images. Subsequently, the corresponding slice was selected on synthetic T1-weighted images. A freehand region of interest (ROI) was placed on the synthetic T1-weighted image while being cross-referenced to the contrast-enhanced images, and with the radiologist being blinded to clinical and pathological information. A freehand ROI was placed just within the inner border of the tumor, in order to avoid the inclusion of surrounding fat. Mean R1, mean R2, and mean PD shown on the viewers were recorded. Mean T1 and mean T2 were calculated based on the following equation: T1 = 1/R1, T2 = 1/R2. Dedicated analysis software automatically calculated apparent diffusion coefficient maps. The same radiologist measured the mean ADC of breast cancers in a similar manner with freehand ROIs set on DWI. The ROIs were copied to the ADC maps to obtain the ADC value.

### 2.4. Pathological Examination

All pathological specimens were evaluated by our institution’s pathology department using their standard clinical protocol. Pathologists evaluated the specimens on the basis of the World Health Organization histological classification of breast tumors [22]. The expressions of ER and PgR were assessed by immunohistochemical staining. Per our institutional protocol, any expression of ER or PgR greater than 1% was considered positive [23,24]. Ki-67 immunoreactivity was evaluated by using the percentage of immunoreactive tumor cells [25]. Based on Ki-67, cancers were divided into a low-proliferation group (Luminal A) (Ki-67 < 14) and a high-proliferation group (Luminal B) (Ki-67 ≥ 14) [3,4].

### 2.5. Statistical Analysis

Data were analyzed using MedCalc 19.8 (MedCalc). All data are expressed as means ± standard deviation, unless otherwise specified. Normal distribution of the T1, T2, PD, and ADC values was tested using the Shapiro–Wilk test. Age, tumor diameter, Ki-67, and the quantitative MRI parameters regarding ER status (positive vs. negative) and Luminal types (A vs. B) were compared using the unpaired *t* test (for normally distributed data) or the Mann-Whitney U Test (for not normally distributed data). Quantitative MR parameters including ADC, were correlated with Ki-67 using a Pearson’s correlation analysis (for normally distributed data) or a Spearman correlation analysis (for not normally distributed data). Among resected tumors, the correlation of quantitative MR values with Ki-67 of both biopsy specimen and surgical specimens was assessed.

The effectiveness of quantitative MR parameters, including ADC, in differentiating the ER-negative group from the ER-positive group and Luminal B from the Luminal A was evaluated using receiver operating characteristic (ROC) analysis. For sensitivity and specificity, the optimal cut-off point was determined using the Youden index. The area under the ROC curve (AUC) was expressed as a mean and had a 95% confidence interval. All statistical tests were two-tailed and significance was set at a *p* < 0.05.

## 3. Results

### 3.1. Patients

Thirty patients were invited, however, two of them refused to participate in this study. Finally, 28 female patients participated in the study (mean age 63.3 ± 12.2 years, range 32–80 years). In one of these 28 patients, bilateral breast cancers were found. Therefore, a total of 29 breast cancers were analyzed. Eighteen patients underwent surgery and their surgical specimens were used for pathological analysis. The other 10 patients underwent preoperative chemotherapy and their biopsy specimens were used for pathological analysis, while tumor size was measured on contrast-enhanced MRI. The mean tumor size was 28 mm (range 12–70 mm). The histological classification of the tumors was as follows; 26 invasive carcinomas of no special type, 2 invasive lobular carcinomas, and 1 invasive micropapillary carcinoma. Most of the tumors were ER-positive (88%) and all of the PgR-positive tumors also showed ER positivity. By combining immunohistochemical marker analysis, 10 (34%) Luminal A tumors, 13 (45%) Luminal B tumors, and 6 (21%) ER-negative tumors were identified.

### 3.2. Relationship with ER Status

**Figure 1:**
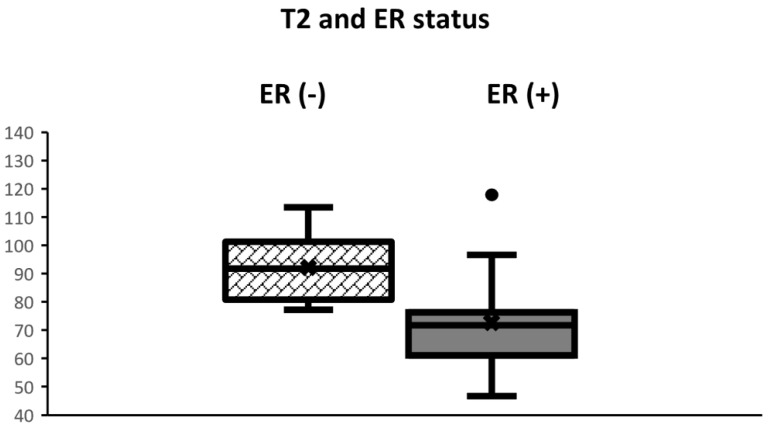
Boxplots displaying the distribution of T2 values according to ER status. The box extends from the 25th to the 75th percentile. The middle horizontal line represents the median value. T2 of ER-negative cancers was significantly higher than that of ER-positive cancers (*p* < 0.01).

**Figure 2:**
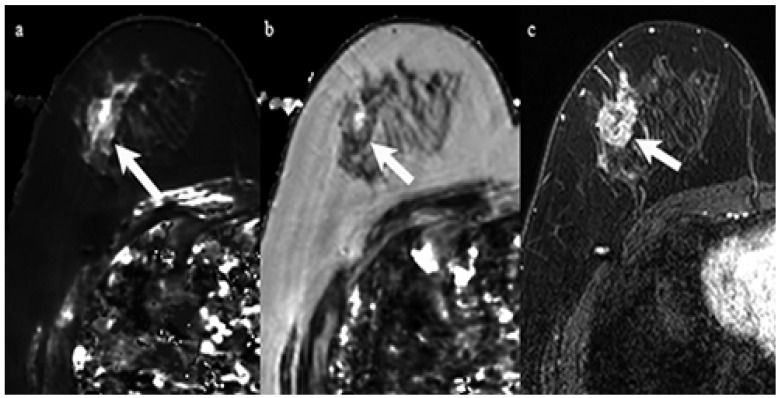
A 59-year-old woman with invasive ductal carcinoma in the right breast. Immunohistochemical staining showed negativity for estrogen receptor and Ki-67 of 60%. (**a**) T1 map shows a high signal mass. Mean T1 of the mass was 1477 msec. (**b**) T2 map shows a high signal mass. Mean T2 of the mass was 96 msec. (**c**) Corresponding contrast enhanced MR image shows a mass with heterogeneous enhancement.

**Figure 3:**
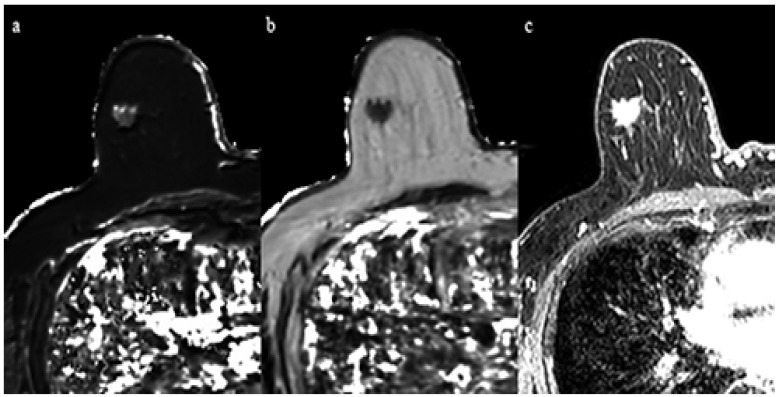
A 69-year-old woman with invasive ductal carcinoma in the right breast. Immunohistochemical staining showed positivity for estrogen receptor and Ki-67 of 10%. (**a**) T1 map shows an intermediate signal mass. Mean T1 of the mass was 986 msec. (**b**) T2 map shows a low signal mass. Mean T2 of the mass was 61 msec. (**c**) Corresponding contrast enhanced MR image.

Clinical and pathological characteristics, and quantitative MRI values for the 29 lesions are shown in Table 1. No statistical significance was found between the ER-positive cancers and the ER-negative cancers regarding age and tumor diameter. The Ki-67 of ER-negative cancers was significantly higher than that of ER-positive cancers (*p* < 0.05). Mean T2 relaxation of ER-negative cancers (92.1 ± 13.0 msec) was significantly higher than that of ER-positive cancers (72.7 ± 15.7 msec, *p* = 0.01) (Figure 1, Figure 2 and Figure 3). Mean T1 relaxation and PD of ER-negative cancers were higher than those of ER-positive cancers, but no significant difference was observed between them. The AUCs of T1, T2, and PD for the differentiation of ER-negative cancers from ER-positive cancers were 0.74 (95% confidence interval (CI): 0.54–0.88), 0.87 (95% CI: 0.69–0.97), and 0.62 (95% CI: 0.42–0.79), respectively. The T2 value had the highest AUC, followed by T1 and PD. The optimal sensitivity and specificity of the T2 value were 100% and 78%.

### 3.3. Relationship with Ki-67

**Figure 4:**
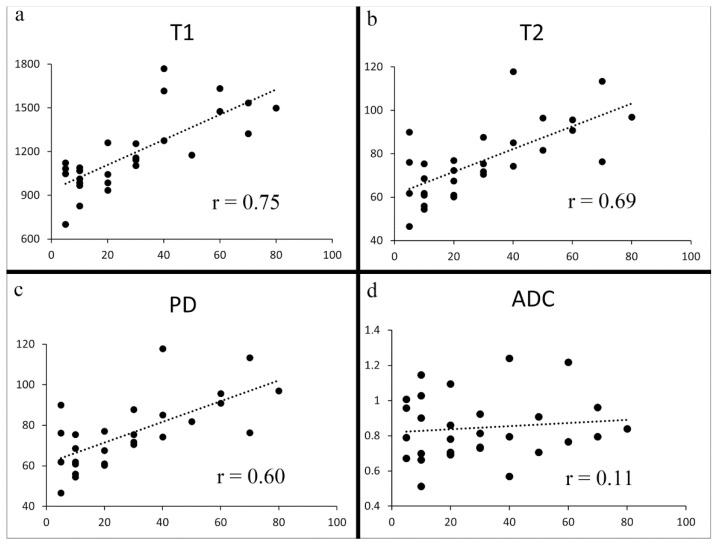
Scatter plots of Ki-67 vs. T1 (**a**), T2 (**b**), PD (**c**), and ADC (**d**). Significant correlations were observed between Ki-67 and T1, between Ki-67 and T2, and between Ki-67 and PD (**a**–**c**) (*p* < 0.001). No significant correlation was seen between Ki-67 and ADC (*p* = 0.57) (**d**).

The Shapiro–Wilk test showed that the T1, T2, PD, and ADC values followed a normal distribution. The T1, T2, and PD derived from synthetic MRI demonstrated a strong positive correlation with Ki-67 (Pearson’s r = 0.75, 0.69, and 0.60 respectively; *p* < 0.001) (Figure 4 and Table 2). Among 19 resected cancers, the correlation of biopsy specimen Ki-67 and resected specimen Ki-67 are demonstrated in Table 2. No significant difference was observed between them. No significant correlation was observed either between ADC and Ki-67 (Pearson’s r = 0.11; *p* = 0.57).

**Figure 5:**
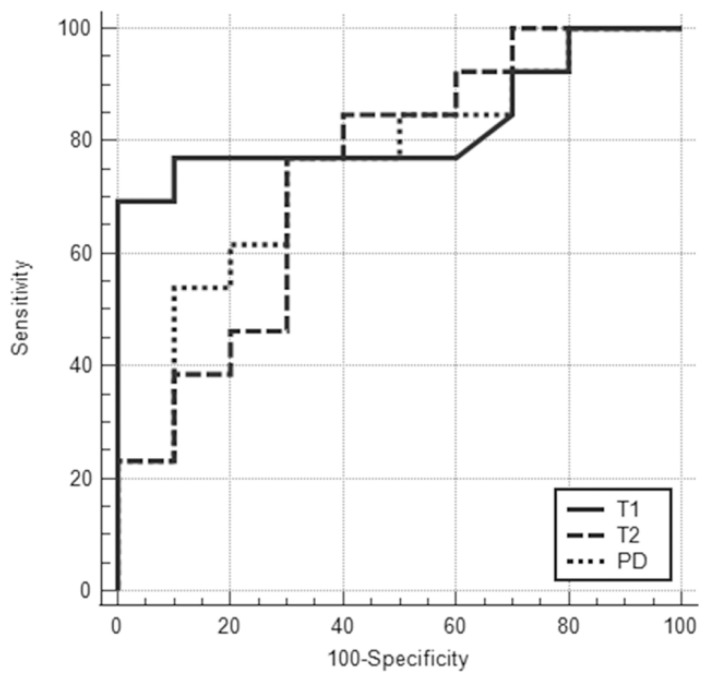
ROC curves for T1, T2, and PD values for differentiating Luminal A from Luminal B cancers showed that the areas under the ROC curve were 0.83 (95% CI: 0.61–0.95), 0.75 (95% CI: 0.52–0.90), and 0.75 (95% CI: 0.53–0.91), respectively.

The clinical and pathological characteristics and quantitative MRI values of ER-positive cancers (n = 23) are shown in Table 3. No statistical significance was found between Luminal A and Luminal B cancers regarding tumor diameter. All T1 (991 ± 132 msec vs. 1259 ± 265 msec), T2 (65.2 ± 12.6 msec vs. 78.5 ± 15.8 msec), and PD values (63.4 ± 11.4% vs. 73.9 ± 12.0%) of Luminal A cancers were significantly lower than those of Luminal B cancers (*p* < 0.05). The AUCs of T1, T2, and PD for the differentiation of Luminal B cancers from Luminal A cancers were 0.83 (95% CI: 0.61–0.95), 0.75 (95% CI: 0.52–0.90), and 0.75 (95% CI: 0.53–0.91), respectively (Figure 5). The T1 value had the highest AUC, followed by T2 and PD. The optimal sensitivity and specificity of the T1 value were 69% and 100%.

## 4. Discussion

Recent advances in MRI have enabled the acquisition of both MR images and quantitative MRI data within a single scan [11,26,27,28,29,30]. In this study, mean T2 relaxation time of ER-negative cancers was significantly higher than that of ER-positive cancers. This result is in good agreement with prior studies [26,27,28,30].

Three previous studies have evaluated the relationship between Ki-67 and quantitative MRI data; however, their results are discordant [26,27,28,29]. T1 and T2 values of Luminal A cancers were significantly lower than those of Luminal B cancers in the present study, which is hypothesized to be due to less neovascularization in luminal A cancers. This result is consistent with three prior studies [26,27,28], but not consistent with Matsuda et al. [29]. Due to the risk of partial volume effects, only patients with lesions larger than 15 mm were analyzed in the present study, while the mean size of breast cancers in Matsuda et al.’s study was 17 mm in Luminal A type and 20 mm in Luminal B type [29]. We speculate that partial volume effects of surrounding tissue, such as fat, might have affected Matsuda et al.’s results [29].

This study showed T1, T2, and PD values, as derived from synthetic MRI, to be significantly and strongly correlated with Ki-67. Ki-67 is considered to represent tumor proliferation status, with a higher Ki-67 being associated with an adverse clinical outcome [2]. This finding has not been previously reported. We believe that minimization of partial volume effects by selecting tumors larger than 15 mm might have allowed us to obtain this result. Rakow-Penner et al. reported that the T1 value of glandular tissue differed approximately 12% between the normal scan and the fat suppressed image due to partial volume effect [31], which could obscure the above-mentioned correlation.

In previous studies, triple negative breast cancers (a subgroup of ER-negative breast cancers) were reported to have a high signal intensity on T2-weighted images due to necrosis [13,14,15,16,20]. A correlation between high signal intensity on T2-weighted images and tumor grade in other subtypes of breast cancers has also been reported. In those studies, it was postulated that edema with corresponding high signal intensity on T2-weighted images was possibly induced by angiogenesis [17,18,19,21,32,33]. Increased water content related to necrosis or edema may cause T1, T2, and PD elongation [34]. Our finding of higher T1 values, higher T2 values, and higher PD values in highly proliferative cancers are consistent with these prior qualitative studies [13,14,15,16,17,18,19,20].

The relationship between Ki-67 and tumor grade with the ADC value has been reported by some previous studies [8,9,14,17,29,35,36]. In the present study, however, there was no significant correlation between ADC and Ki-67. This finding is similar to prior studies [8,9,17]. Kim et al. reported that highly aggressive tumors may outgrow the oxygen supply of their vascular system, resulting in necrosis and decreased cellularity [17,37]. In high grade tumors, a higher tumor grade often goes hand in hand with a higher ADC value [14,17,36]. In contrast, in relatively well-differentiated tumors, moderately proliferative cancers tend to have a higher cellularity than low proliferative tumors and generally have a lower ADC [35]. This paradoxical phenomenon can cause confusion in subtype prediction based on DWI. Furthermore, both high cell density areas, hematoma, and fat show low ADC values, and discriminating them can be challenging. Contrary to the ADC, all T1, T2, and PD values of high proliferative tumors were higher than those of low proliferative tumors. Since the T1, T2, and PD value assessments did not experience this paradoxical phenomenon, we believe they are more useful than ADC measurements in this setting.

There are several limitations in this study. First, the number of patients in this study was relatively small. Second, 10 out of 28 patients underwent neoadjuvant chemotherapy and only their biopsy specimens could be used for analysis. A significant difference in Ki-67 has been reported between specimens obtained at preoperative biopsy and surgery [38]. However, breast cancer patients with very high Ki-67 values very often undergo preoperative chemotherapy, and surgical specimens without previous chemotherapy are hardly ever obtained [39]. In addition, in the 19 cancers for which both surgical and biopsy specimens were available, the correlation coefficients of quantitative MRI values versus Ki-67 were similar between surgical and biopsy specimens. Third, because of the risk of partial volume effects on quantitative MRI measurements, only large mass lesions were included in this study. Our finding may not be applicable to non-mass lesions or smaller lesions, such as ductal carcinoma in situ [29]. Fourth, all patients underwent biopsy before MRI. Hemorrhage induced by biopsy might have affected the measured quantitative values.

## 5. Conclusions

In conclusion, quantitative values derived from synthetic MRI show potential for subtyping of invasive breast cancers.

## Figures and Tables

**Table 1:** Characteristics of cancers according to ER status.

	ER ^1^-Positive Cancers (n = 23)	ER ^1^-Negative Cancers (n = 6)	*p* Value
Age (y)	64.0 ± 13.2	60.7 ± 8.0	0.35
Diameter (mm)	28.5 ± 16.3	27.7 ± 9.8	0.79
Ki-67	23.9 ± 18.6	51.7 ± 23.2	0.01
T1 (msec)	1142 ± 253	1331 ± 201	0.08
T2 (msec)	72.7 ± 15.7	92.1 ± 13.0	0.006
PD ^2^ (%)	69.3 ± 12.7	74.5 ± 5.8	0.39

Data are presented as mean ± standard deviation. ^1^ ER, estrogen receptor; ^2^ PD, proton density.

**Table 2:** Correlation of quantitative MR values with Ki-67.

			Pearson’s Coefficient	*p* Value
All (n = 29)		T1	0.75	<0.0001
		T2	0.69	<0.0001
		PD ^1^	0.60	0.0006
		ADC ^2^	0.11	0.57
Resected cancers (n = 19)	Biopsy Ki-67 (n = 19)	T1	0.72	0.0005
		T2	0.74	0.0003
		PD^1^	0.57	0.01
	Resected specimen Ki-67 (n = 19)	T1	0.74	0.0003
		T2	0.72	0.0005
		PD^1^	0.59	0.008

^1^ PD, proton density; ^2^ ADC, apparent diffusion coefficient.

**Table 3:** Characteristics of Luminal A and Luminal B cancers.

	Luminal A Group (n = 10)	Luminal B Group (n = 13)	*p* Value
Age (y)	72.0 ± 9.1	57.9 ± 12.5	0.005
Diameter (mm)	28.0 ± 15.5	28.8 ± 16.9	0.90
Ki-67	8.0 ± 2.6	36.2 ± 16.1	0.001
T1 (msec)	991 ± 132	1259 ± 265	0.008
T2 (msec)	65.2 ± 12.6	78.5 ± 15.8	0.047
PD ^1^ (%)	63.4 ± 11.4	73.9 ± 12.0	0.041

Data are presented as mean ± standard deviation. ^1^ PD, proton density

## Data Availability

Datasets used and/or analyzed during the current study are available from the corresponding author upon reasonable request.
